# Biostimulants in the Formation of *Spondias tuberosa* Rootstocks

**DOI:** 10.3390/plants15213351

**Published:** 2026-10-29

**Authors:** Bruno Silva Guirra, Francisco Thiago Coelho Bezerra, Rejane Maria Nunes Mendonça, Maria Alaíne da Cunha Lima, Jandira Pereira da Costa, Walter Esfrain Pereira, Marlene Alexandrina Ferreira Bezerra, Adailson Tulio dos Santos Silva, Keylan Silva Guirra, José Eduardo Santos Barboza da Silva, Gleyse Lopes Fernandes de Souza

**Affiliations:** 1 Center for Agricultural Sciences, Federal University of Paraíba, Areia 58397-000, PB, Brazil; 2 Course of Agronomy, Ibiapaba Campus, State University Vale do Acaraú, São Benedito 62370-000, CE, Brazil; 3 Independent Researcher, São Benedito 62370-000, CE, Brazil; 4 Coordination of Educational Practice Units, Federal Institute of Education, Science and Technology of Baiano, Xique-Xique 47400-970, BA, Brazil

**Keywords:** *Umbu* tree, biostimulants, chlorophyll fluorescence, gas exchange, seedling quality, Caatinga

## Abstract

The *umbu* tree (*Spondias tuberosa* Arruda) is a fruit-bearing species native to the semiarid region of Brazil that possesses high ecological and socioeconomic value; however, its cultivation remains limited by a lack of technologies for producing high-quality seedlings. In this context, the use of biostimulants offers a strategy to optimize seedling growth and physiological efficiency. This study aimed to evaluate the effects of different biostimulant sources and dosages on the growth, physiology, and quality of *S. tuberosa* seedlings. The experiment followed a 2 × 6 factorial design, with two sources (Bio-I and Bio-II) and six concentrations (0.0, 0.5, 1.5, 2.5, 3.5, and 4.5 mL L^−1^) of biofertilizer, arranged in a randomized block design. The evaluations included biometric variables, dry biomass, the Dickson quality index, leaf chlorophyll indices, chlorophyll fluorescence, and gas exchange. Leaf chlorophyll indices and gas exchange parameters were not significantly affected, indicating physiological stability. Intermediate doses (1.7–1.9 mL L^−1^) tended to increase the Fv/Fo and Fv/Fm values; however, these responses should be interpreted as descriptive trends since no significant treatment effect was detected. Height growth, shoot biomass accumulation, and the Dickson quality index increased with dosage, peaking at 4.5 mL L^−1^, whereas leaf production was greater with Bio II. Multivariate analysis indicated that leaf temperature played a major role in the growth characteristics and physiological processes evaluated. Lower leaf temperatures were associated with greater stomatal limitation of CO_2_ exchange and internal carbon accumulation. Foliar application of Bio I and Bio II improved the growth and quality of *S. tuberosa* seedlings, with the highest responses recorded for plant height and specific biometric traits at 4.5 mL L^−1^ within the evaluated concentration range.

## 1. Introduction

The *umbu* tree (*Spondias tuberosa* Arruda) is a fruit-bearing tree native to and endemic to the Caatinga; it is recognized for its high hardness and broad adaptability to the low water availability characteristic of the Brazilian semiarid region [1,2]. It is a small- to medium-sized species featuring a short, gnarled trunk and grayish bark; it is also deciduous, shedding its leaves during the driest periods as a strategy to minimize water loss [3,4]. Its survival in semiarid environments is strongly linked to the presence of a xylopodium, a specialized underground structure for storing water, carbohydrates, and nutrients, which enables the tree to withstand prolonged droughts and resprout when rains return [5,6]. The fruit is a drupe with succulent, sweet-and-sour pulp; it is widely consumed fresh and processed into pulp, sweets, jams, juices, popsicles, and ice cream while also containing bioactive compounds of nutritional and functional interest [7,8,9].

From a production standpoint, fruiting occurs predominantly between January and April, and the exploitation potential of this species has sparked growing interest because of its integration into more sustainable and diversified production systems [1,10]. In this context, the *umbu* tree stands out as a strategic component for agroforestry systems, Caatinga enrichment initiatives, and the strengthening of family farming in the Brazilian semiarid region owing to its ecological and socioeconomic value [2,7,11,12]. Despite this potential, cultivation of the species remains limited by its low level of domestication and reliance on wild harvesting, which is a consequence of the lack of specific crop management technologies [13]. Key obstacles to establishing commercial orchards include the availability of high-quality seedlings and a long juvenile period; plants propagated from seeds may only begin producing fruit after approximately ten years, whereas grafted seedlings tend to enter the productive phase much earlier, at an average age of 4.5 years [14,15,16].

Thus, producing vigorous, uniform seedlings with well-developed root systems is crucial not only for initial field establishment but also for obtaining rootstocks for grafting, an essential strategy for accelerating the onset of production and increasing the efficiency of cultivation systems [17,18,19]. Consequently, incorporating biostimulants into nursery practices can optimize physiological and morphological parameters, increasing the production of uniform batches suitable for grafting.

The use of plant biostimulants is a promising alternative for optimizing the initial growth, rooting, and uniformity of perennial fruit tree seedlings. In combination with plant regulators, amino acids, vitamins, humic acids, and microbial extracts, these products modulate physiological and metabolic processes, promoting enhanced vegetative growth, leaf expansion, biomass accumulation, and root system development [20,21]. Among these biostimulants, some are predominantly composed of growth regulators, such as Bio I, whereas others combine plant regulators with seaweed extracts, mineral nutrients, and betaine, such as Bio II; both of these methods were used in this study. Differences in composition can lead to distinct responses among products, even when they belong to the same category. These effects are particularly relevant during the nursery stage, as they facilitate the production of uniform seedlings that are structurally suitable for grafting.

In *S. tuberosa*, the foliar application of growth regulators, such as gibberellic acid (GA_3_), has been shown to increase vegetative growth and seedling biomass, with responses depending on the applied dose. Pires et al. [14] reported maximum biomass production with the application of approximately 337 mg of GA_3_ L^−1^, highlighting the potential of this compound to accelerate early growth and reduce the time required for rootstock formation. Similarly, commercial biostimulants formulated from bioactive compounds, including plant growth regulators (auxins, gibberellins, and cytokinins), amino acids, organic substances, and seaweed extracts, have positive effects on early growth, root development, and the morphophysiological characteristics of various crops, although the results vary according to the species and the applied dose [22,23].

Despite these advances, studies comprehensively evaluating the effects of biostimulants on the growth, physiology, and quality indicators of *S. tuberosa* seedlings, particularly those linking biometric and physiological variables to rootstock formation potential, remain scarce. This gap limits the understanding of the mechanisms by which these products can contribute to the production of more vigorous and uniform seedlings, as well as the development of management strategies to optimize rootstock formation for grafting.

Given this context, the application of biostimulants may increase the growth and quality of *S. tuberosa* rootstocks by modulating the physiological and metabolic processes associated with plant development. These compounds can stimulate cell division and elongation, increase photosynthetic efficiency, improve water and nutrient uptake and utilization, and increase root system development and biomass accumulation. Consequently, there is a practical need to shorten the production cycle of graft-compatible rootstocks, thereby making the process more efficient and sustainable. The expected outcome is the production of more vigorous, uniform seedlings with superior quality metrics, reducing the time required to produce rootstocks ready for grafting. Thus, this study aimed to evaluate the effects of different sources and dosages of biostimulants on the growth, physiological characteristics, and quality of *Spondias tuberosa* Arruda rootstocks.

## 2. Results

### 2.1. Growth Responses

**Figure 1:**
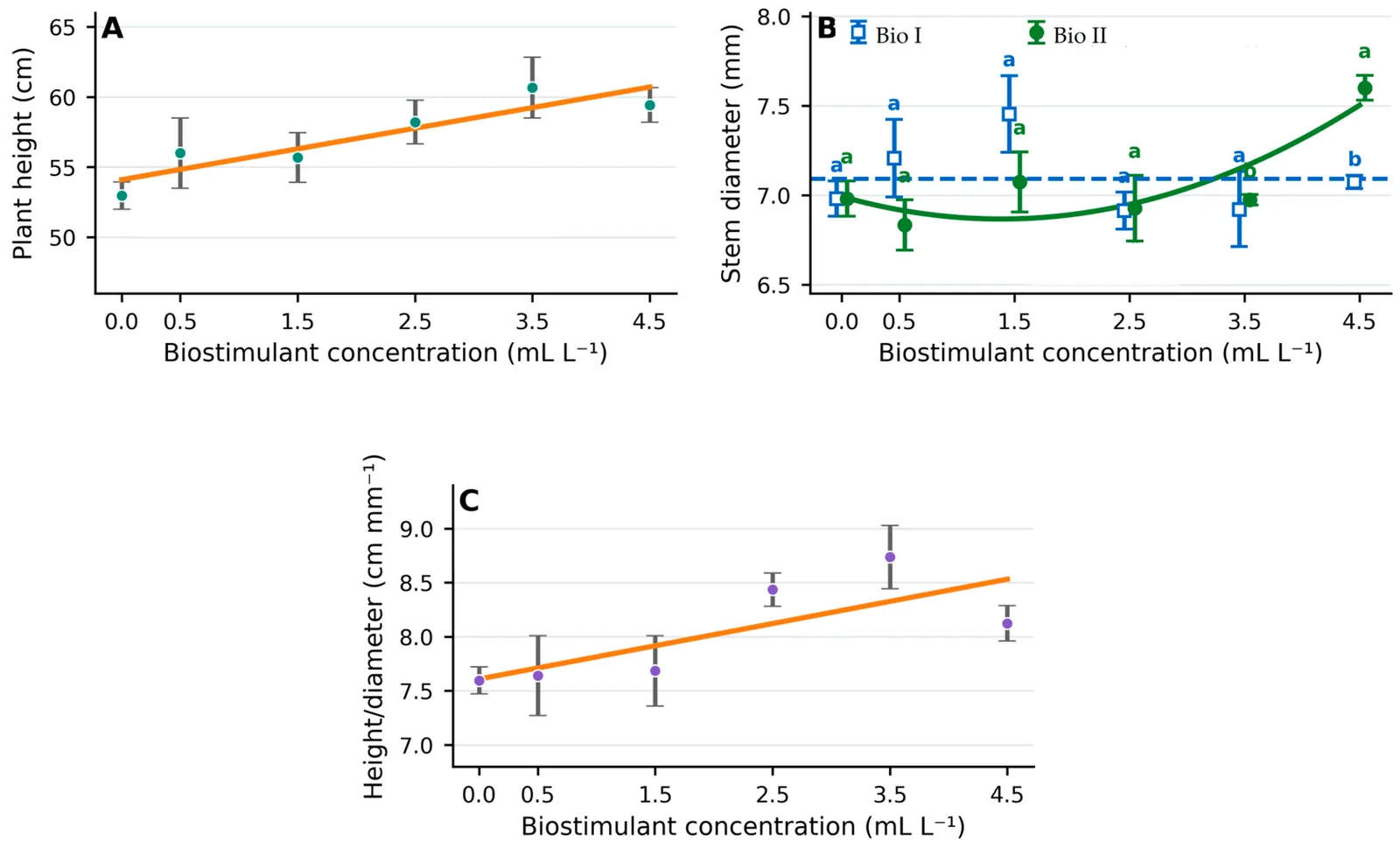
Mean values ± standard errors of the means (I) for the growth of *Spondias tuberosa* Arruda rootstocks in relation to the source and/or concentration of biostimulants. (**A**) Plant height (ŷ = 54.10 + 1.4672**x; R^2^ = 0.83); (**B**) Stem diameter at the substrate level for Bio I (ŷ = 7.09; concentration effect = ns) and Bio II (ŷ = 6.99 − 0.1812**x + 0.0655*x^2^; R^2^ = 0.75); (**C**) Relationship between height growth and stem diameter (ŷ = 7.61 + 0.2051**x; R^2^ = 0.57). Mean ± SE. In (**B**), letters compare products within concentrations (*t* test, *p* ≤ 0.05). ^†^ *p* < 0.10; * *p* < 0.05; ** *p* < 0.01. Coefficients without codes are not significant.

The height growth of *Spondias tuberosa* rootstocks was influenced exclusively by the concentration of the biostimulants (F = 3.33; *p* = 0.0216; CV = 6.60%), with an estimated increase of 1.47 cm for every 1 mL L^−1^ increase in product concentration (Figure 1A). In the absence of biostimulants, the estimated rootstock height was 54.10 cm, increasing to 60.70 cm at a dose of 4.5 mL L^−1^, an increase of 12.2%.

With respect to the stem diameter of the rootstocks at the substrate level, an interaction was observed between the biostimulant source and concentration (F = 3.42; *p* = 0.0196; CV = 3.13%). However, a regression model fit was observed only for Bio II, with an increase in rootstock diameter starting at a concentration of 1.4 mL L^−1^ and increasing from 6.86 mm to 7.50 mm at a concentration of 4.5 mL L^−1^ (Figure 1B). Conversely, rootstocks treated with Bio I presented a mean diameter of 7.09 mm, representing a 5.5% reduction compared with the largest diameter obtained with Bio II.

The relationship between height growth and stem diameter growth was influenced only by the biostimulant concentration (F = 4.76; *p* = 0.0043; CV = 6.04%), with the lowest ratio (7.61 cm mm^−1^) estimated for the treatment without biostimulant application and the highest ratio (8.53 cm mm^−1^) for the 4.5 mL L^−1^ concentration (Figure 1C).

The number of leaves on the rootstocks was influenced only by the biostimulant source (F = 4.59; *p* = 0.0434; CV = 7.83%), with no fit to a regression model; the highest mean was 23.3 ± 0.5 leaves with the application of Bio II, and the lowest mean was 22.0 ± 0.4 leaves with Bio I.

### 2.2. Biomass Accumulation and Seedling Quality

For the variables root dry matter mass (F ≤ 2.04; *p* ≥ 0.1127; CV = 21.22%), shoot dry matter mass (F ≤ 2.48; *p* ≥ 0.0627; CV = 18.67%), total dry matter mass (F ≤ 2.27; *p* ≥ 0.0830; CV = 19.67%), and the ratio between shoot dry matter mass and root dry matter mass (F ≤ 1.48; *p* ≥ 0.2367; CV = 16.87%), no significant effects were observed regarding the interaction between sources and concentrations, nor for the factors evaluated in isolation, with mean values of 13.25 ± 0.52 g, 2.97 ± 0.10 g, 16.22 ± 0.60 g, and 0.23 ± 0.04 g g^−1^, respectively.

**Figure 2:**
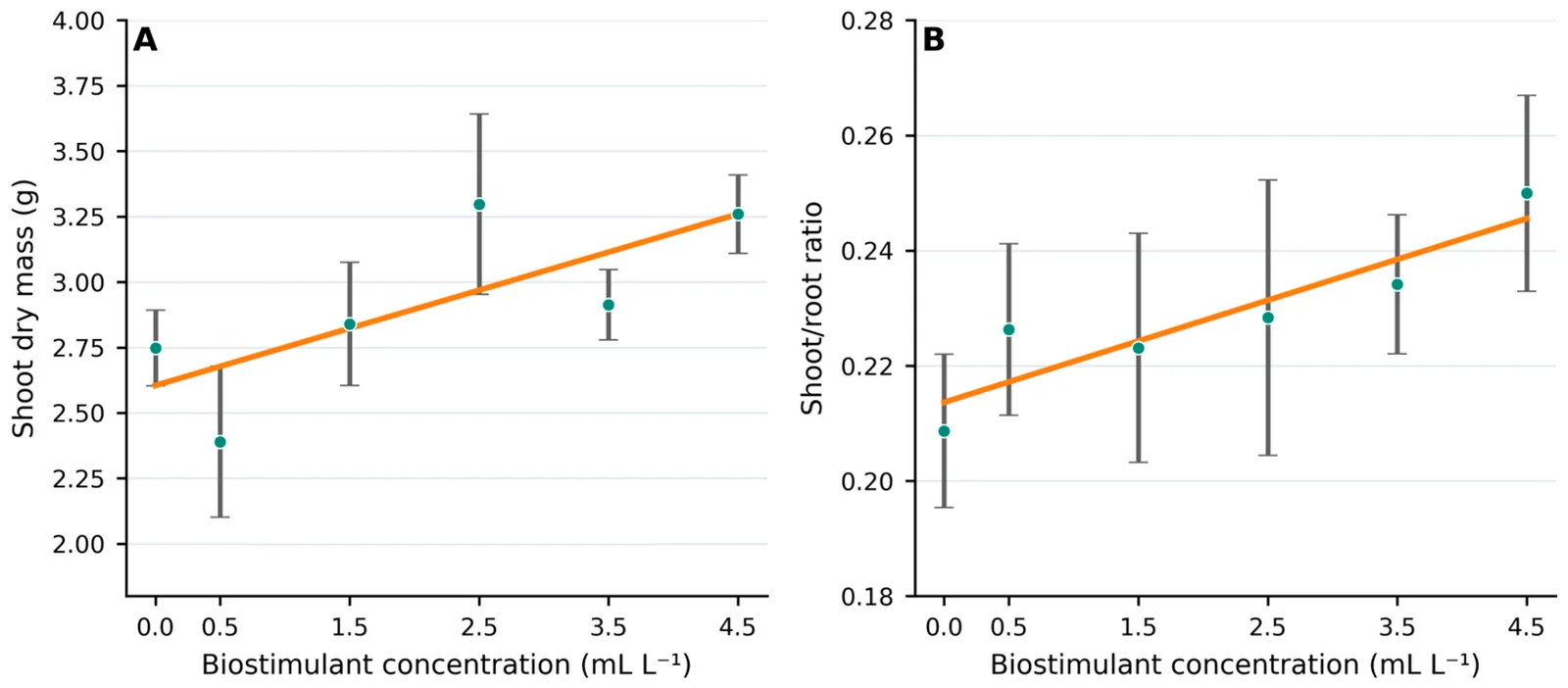
Mean values ± standard errors of the means (I) for the biomass production of *Spondias tuberosa* Arruda rootstocks as a function of the biostimulant concentration. (**A**) Shoot dry matter mass (ŷ = 2.60 + 0.1455 *x; R^2^ = 0.56); (**B**) ratio of shoot dry mass to root dry mass (ŷ = 0.21 + 0.0071^†^x; R^2^ = 0.83). Mean ± SE. ^†^ *p* < 0.10; * *p* < 0.05; ** *p* < 0.01. Coefficients without codes are not significant.

However, an increase in shoot dry matter mass was estimated as the biostimulant concentration increased from 2.60 g without application to 3.25 g at 4.5 mL L^−1^, an increase of 0.65 g, equivalent to a 25.0% gain, or 0.15 g (5.8%) for each unit increase in biostimulant concentration (Figure 1A). The ratio of shoot dry matter mass to root dry matter mass also increased with increasing biostimulant concentration, from 0.21 g g^−1^ without the biostimulant to 0.24 g g^−1^ at 4.5 mL L^−1^, an increase of 14.3% (Figure 2B).

The Dickson quality index (DQI) was influenced by the concentration of the biostimulants (F = 2.91; *p* = 0.0365; CV = 18.72%). However, it was not possible to fit a mathematical model capable of adequately describing the variable’s behavior in relation to the doses evaluated, with a mean of 1.96 ± 0.08.

Overall, ANOVA (*p* ≤ 0.05) revealed significant effects for root dry mass, total dry mass, and the Dickson quality index in some instances. However, it was not possible to fit significant regression models; consequently, the results were interpreted merely as discrete significant differences rather than as continuous responses to the biostimulant concentrations.

### 2.3. Photosynthetic Pigments and Chlorophyll Fluorescence

Foliar indices for chlorophyll a (F ≤ 1.14; *p* ≥ 0.3677; CV = 3.94%), chlorophyll b (F ≤ 1.54; *p* ≥ 0.2271; CV = 15.53%), and total chlorophyll (F ≤ 1.39; *p* ≥ 0.2656; CV = 5.87%), as well as the chlorophyll a/b ratio (F ≤ 3.15; *p* ≥ 0.0899; CV = 15.82%) in *S. tuberosa* rootstocks, were not influenced by the sources or concentrations of the biostimulants—either individually or through the interaction between these factors—nor did they fit a regression model. The observed mean values were 31.71 ± 0.20 for chlorophyll a, 9.07 ± 0.24 for chlorophyll b, 40.77 ± 0.40 for total chlorophyll, and 3.67 ± 0.10 for the chlorophyll a/b ratio.

**Figure 3:**
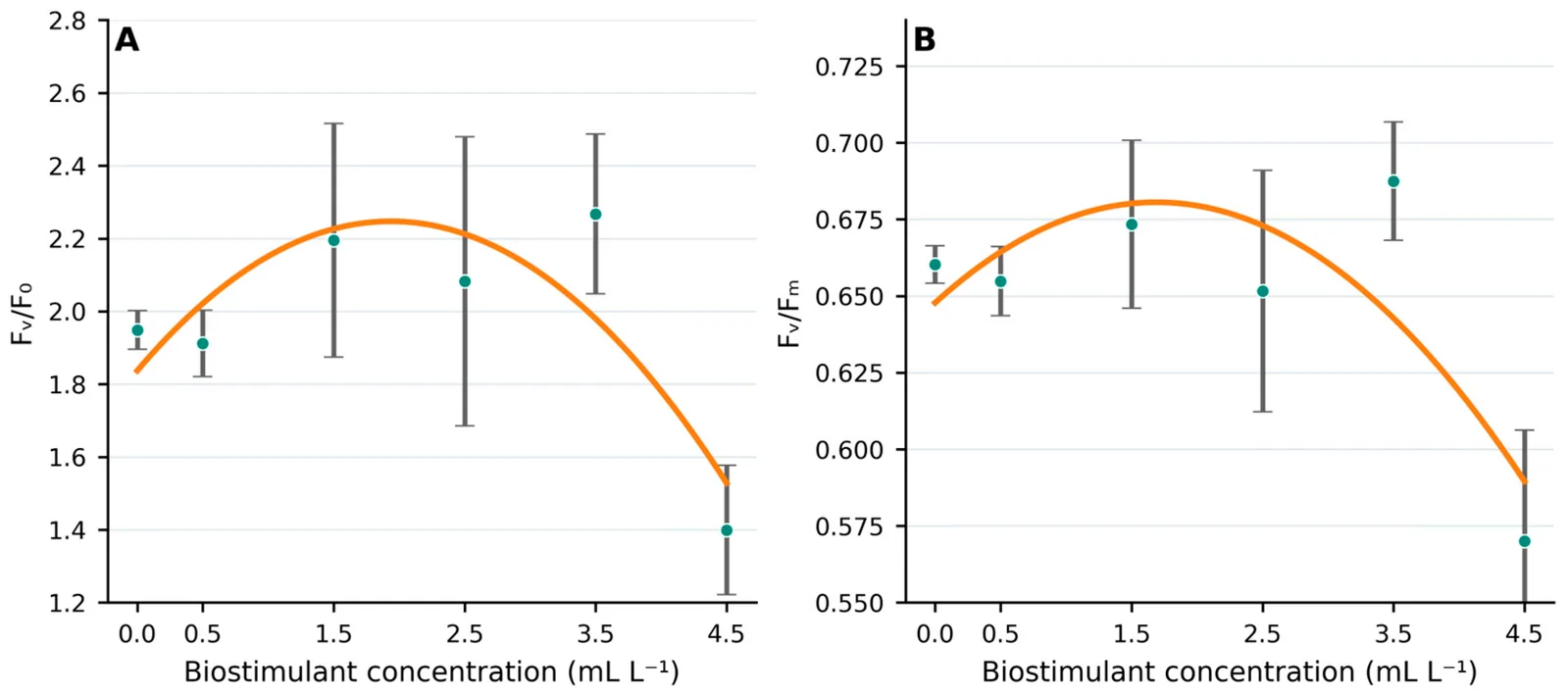
Mean values ± standard errors of the means (I) for chlorophyll a fluorescence of *Spondias tuberosa* Arruda rootstocks as a function of biostimulant concentration. (**A**) Potential relationship between photochemical and nonphotochemical processes (ŷ = 1.84 + 0.4236x − 0.1094*x^2^; R^2^ = 0.71); (**B**) potential quantum efficiency of photosystem II (ŷ = 0.65 + 0.0388^†^x − 0.0115*x^2^; R^2^ = 0.63). Mean ± SE. ^†^ *p* < 0.10; * *p* < 0.05; ** *p* < 0.01. Coefficients without codes are not significant.

Similarly, chlorophyll fluorescence variables, including initial fluorescence (Fo) (F ≤ 0.67; *p* ≥ 0.4228; CV = 18.52%), maximum fluorescence (Fm) (F ≤ 0.84; *p* ≥ 0.5347; CV = 32.47%), and variable fluorescence (Fv) (F ≤ 1.11; *p* ≥ 0.3838; CV = 42.55%), were unaffected by the factors or their interaction and did not fit a regression model. No significant effects were detected for the ratio of photochemical to nonphotochemical processes (Fv/Fo) (F ≤ 1.48; *p* ≥ 0.2360; CV = 31.75%); however, increasing the biostimulant concentration to 1.9 mL L^−1^ yielded the highest value (2.27), a 23.4% increase compared with that of the control, whereas the value decreased to 1.57 at 4.5 mL L^−1^ (Figure 3A). A similar pattern was observed for photosystem II quantum efficiency (Fv/Fm); however, there were no significant effects from the factors or their interaction (F ≤ 2.47; *p* ≥ 0.0639; CV = 9.88%), and the highest value (0.68) was recorded at a biostimulant concentration of 1.7 mL L^−1^ (a 4.6% increase over the control), whereas the efficiency decreased to 0.59 at 4.5 mL L^−1^.

The fluorescence variables showed quadratic trends, with maximum values at intermediate doses (1.7–1.9 mL L^−1^). However, since the overall effects were not significant according to ANOVA, these trends should be considered merely descriptive, lacking statistical confirmation of a treatment effect.

### 2.4. Gas Exchange

The variables related to gas exchange in the leaves of S. tuberosa rootstocks (leaf tem-perature—T °C, stomatal conductance—Gs, stomatal resistance—Rs, internal CO_2_ concen-tration—Ci, relative stomatal limitation—Ci/Ca, net CO_2_ assimilation—A, transpira-tion—E, water-use efficiency— WUE, instantaneous carboxylation efficiency— CE, and intrinsic water-use efficiency— iWUE were not influenced by the biostimulant sources or concentrations, either individually or through their interaction, nor did these variables fit a regression model.

The *S. tuberosa* rootstocks are expressed as the mean ± standard error of the mean, with values of 37.35 ± 0.27 °C for leaf temperature, 0.06 ± 0.02 mmol H_2_O m^−2^ s^−1^ for stomatal conductance (Gs), 29.82 ± 3.30 s mol^−1^ H_2_O m^−2^ for stomatal resistance (Rs), 155.90 ± 12.42 μmol CO_2_ mol^−1^ air for internal CO_2_ concentration (Ci), 0.41 ± 0.03 for relative stomatal limitation(Ci/Ca), 7.03 ± 0.55 μmol CO_2_ m^−2^ s^−1^ for net CO_2_ assimilation (A), 2.89 ± 0.31 mmol H_2_O m^−2^ s^−1^ for transpiration (E), 3.36 ± 0.37 μmol CO_2_ mol^−1^ H_2_O for water-use efficiency (WUE), 0.03 ± 0.01 mol CO_2_ m^−2^ s^−1^ for instantaneous carboxylation efficiency(CE), and 154.79 ± 15.61 μmol CO_2_ mol^−1^H_2_O for intrinsic water-use efficiency (iWUE).

### 2.5. Multivariate Analyses

To integrate the morphophysiological attributes of *Spondias tuberosa* rootstocks, principal component analysis (PCA) and hierarchical cluster analysis were performed. PCA allowed the dataset’s variability to be summarized into four principal components, which together explained 77.57% of the total observed variance (Table 1).

The first principal component (PC1), accounting for 28.21% of the total variability, demonstrated associations between growth attributes and variables related to seedling gas exchange (Table 2). The greatest contributions (eigenvector^2^ × 100) to this component were observed for leaf temperature (9.79%), relative stomatal limitation (Ci/Ca (9.62%), internal CO_2_ concentration (Ci; 9.56%), number of leaves (6.72%), plant height (6.29%), height/stem diameter ratio (6.22%), transpiration (5.79%), stomatal conductance (5.34%), and water-use efficiency (5.06%). The distributions of these variables suggest that changes in water status and gas exchange dynamics are closely linked to the vegetative growth of the seedlings. Overall, lower leaf temperatures were associated with higher Ci and Ci/Ca values, as well as increased leaf production and height growth, highlighting the integration between physiological processes and plant development.

The second principal component (PC2) explained 23.68% of the total variability and was characterized primarily by variables associated with seedling quality and physiological performance. The highest contributions were observed for the stomatal resistance (10.07%), Dickson quality index (9.22%), root dry mass (8.11%), total dry mass (7.34%), total leaf chlorophyll index (7.34%), net CO_2_ assimilation (6.82%), leaf chlorophyll a (6.32%) and chlorophyll b (6.31%) indices, intrinsic water-use efficiency (5.70%), and water-use efficiency (5.42%). These results demonstrate that attributes related to root biomass accumulation, seedling morphological quality, and photosynthetic capacity were decisive in discriminating between treatments, reinforcing the importance of integrating growth and physiology when defining the quality of the produced plants. Additionally, an inversely proportional relationship was observed between stomatal resistance and net CO_2_ assimilation, reflecting the expected physiological behavior wherein increased stomatal resistance imposes diffusive limitations on carbon entry into the leaves, resulting in lower photosynthetic rates in the seedlings.

Notably, the interpretation of the physiological maximum root biomass should be considered speculative, as it requires validation through further experiments. The Fv/Fm values observed in this study remained within the optimal range for *S. tuberosa*, which is consistent with published data for this species and reinforces the stability of the photosynthetic apparatus under various biostimulant dosages. Furthermore, differences in composition between Bio I (growth regulators) and Bio II (growth regulators combined with seaweed extract, amino acids, and betaine) may have favored more pronounced responses in terms of structural growth and biomass accumulation.

The third principal component (PC3), accounting for 14.36% of the total variability, was strongly associated with the photochemical parameters of chlorophyll fluorescence. The most representative variables were the maximum quantum efficiency of photosystem II, Fv/Fm (15.50%); the variable fluorescence, Fv (14.98%); the ratio between photochemical and nonphotochemical processes, Fv/Fo (13.63%); the maximum fluorescence, Fm (12.96%); the ratio of leaf chlorophyll a/b indices (6.83%); and the leaf chlorophyll b index (6.57%). PC3 was primarily associated with chlorophyll fluorescence variables. Additionally, an inverse relationship was observed between photochemical performance and chlorophyll b indices, suggesting that greater utilization of light energy occurred independently of significant increases in the concentration of this pigment.

The fourth principal component (PC4), accounting for 11.32% of the total variance, highlighted vegetative vigor, with an emphasis on biomass allocation and partitioning to the plant’s aerial structure. The greatest contributions to this component were observed for shoot dry mass (18.40%), the shoot-to-root dry mass ratio (11.55%), total dry mass (8.86%), and stem diameter (8.61%). The clustering of these morphological traits demonstrated that seedlings exhibiting greater stem diameter growth also accumulated more total dry mass. Furthermore, this component highlights a carbon partitioning strategy in which biomass gain is strongly directed toward shoot expansion, explaining the concomitant increase in the shoot-to-root ratio (SDM/RDM), reflecting structural robustness and strategies for allocating photoassimilates to support seedling development.

**Figure 4:**
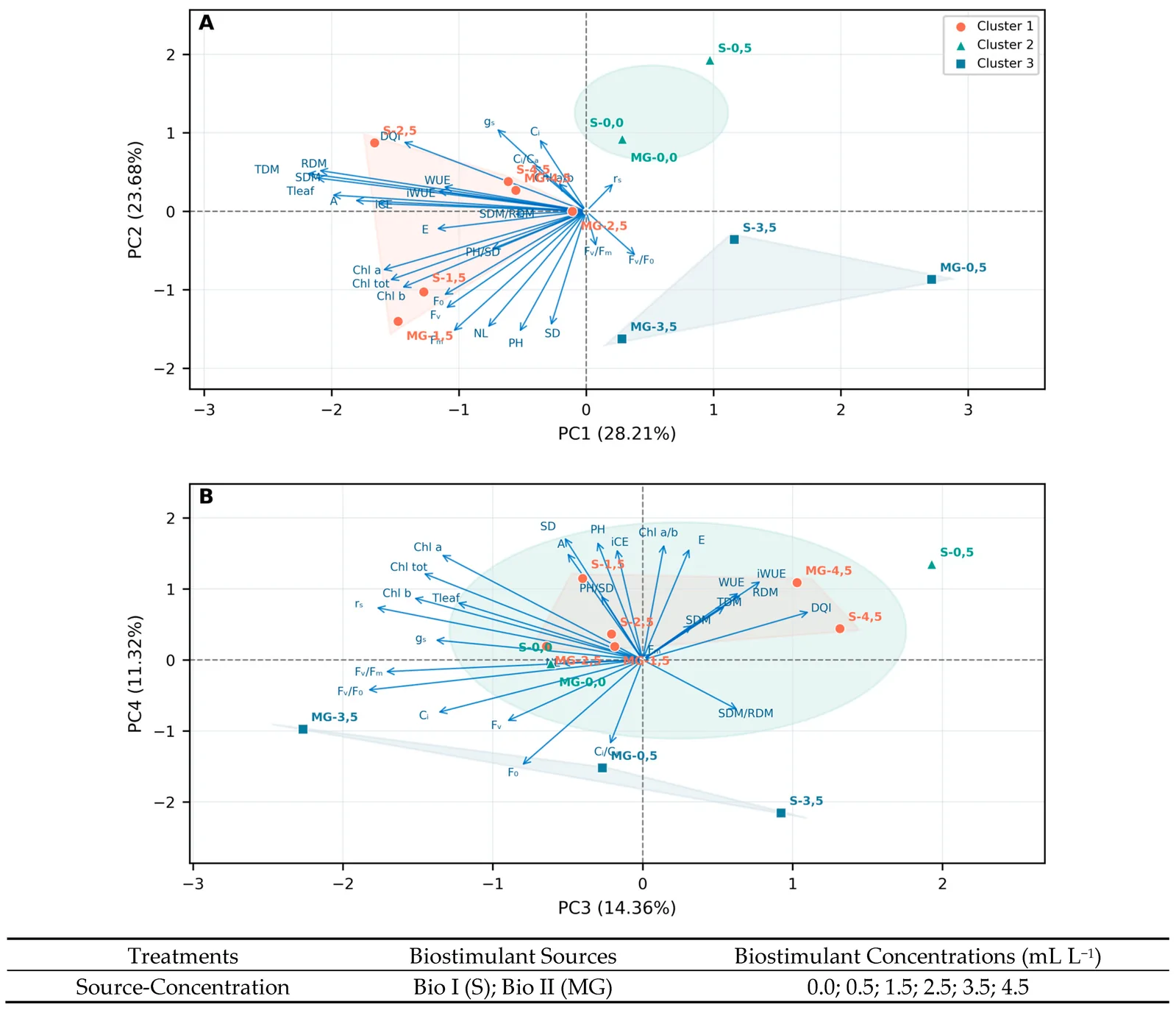
Dispersion of morphophysiological variables in *Spondias tuberosa* Arruda rootstocks and treatment groupings on the basis of scores. (**A**) Clustering based on the scores of the first (PC1) and second (PC2) principal components; (**B**) clustering based on the scores of the third (PC3) and fourth (PC4) principal components. Standardized treatment means. PC1–PC4 explain 77.57% of the total variance; three clusters were obtained via Ward’s method via PC1–PC2 scores. Bio I (S = Stimulate^®^); Bio II (MG = Matriz G^®^).

Cluster analysis, which is based on principal component scores, enabled the formation of three distinct treatment groups (Figure 4). The first group comprised Bio II treatments at concentrations of 1.5, 2.5, and 4.5 mL L^−1^ and Bio I treatments at 1.5 and 2.5 mL L^−1^, as well as the control treatment (no biostimulant), indicating high similarity among these response profiles. The second group consisted of Bio II at a dose of 0.5 mL/L and Bio I at doses of 0.5, 3.5, and 4.5 mL L^−1^. The third group was formed exclusively by the Bio II treatment at a dose of 3.5 mL L^−1^, demonstrating a distinct morphophysiological behavior compared with the other treatments. This separation confirms the ability of biostimulants to integrally modify plant growth and physiological attributes, highlighting the influence of product composition and applied concentration on the overall response of plants.

## 3. Discussion

### 3.1. Effects of Biostimulants on the Growth and Quality of Spondias tuberosa Rootstocks

The growth of *S. tuberosa* seedlings was positively influenced by the application of biostimulants, demonstrating the favorable response of this species to exogenous hormonal regulation during the juvenile phase. This result is particularly relevant to the *umbu tree* production chain, given that slow initial growth is a major constraint on seedling production, rootstock development, and the expansion of commercial cultivation. In this context, the use of biostimulants represents a promising strategy to reduce the time seedlings spend in nurseries and accelerate initial plant establishment, thereby contributing to domestication and commercial-scale cultivation efforts.

The observed effects on growth can be attributed to the integrated action of plant regulators present in the formulations, specifically auxins, gibberellins, and cytokinins, which act complementarily to regulate cell division, cell expansion, cambial activity, vascular differentiation, and vegetative organ development [25,26,27]. In addition to their direct effects on growth, recent studies have demonstrated that biostimulants can increase nutrient use efficiency, stimulate metabolic activity, and strengthen source–sink relationships, thereby promoting greater conversion of photoassimilates into structural biomass [21,28]. Thus, the observed increases in height, stem diameter, and leaf number reflect not only direct hormonal stimulation but also the optimization of physiological processes associated with plant growth.

The observed biometric responses further suggest behavior consistent with the phenomenon of hormesis, characterized by physiological stimulation at low or moderate doses and a reduced response at higher concentrations [29]. This pattern has frequently been reported for biostimulants containing plant regulators, amino acids, and seaweed extracts, where excessive doses can lead to receptor saturation, competition between hormonal signaling pathways, or metabolic imbalances capable of limiting the benefits initially observed [30,31]. Additionally, Rouphael and Colla [21] highlighted that the efficacy of these products depends not only on the applied dose but also on the formulation composition, the stability of bioactive compounds, and the plants’ capacity for absorption and translocation, factors that may explain performance differences among the evaluated biostimulants.

The height–stem diameter ratio (H/SD) was also positively related to biostimulant application. This index is widely used as an indicator of seedling morphological quality because it expresses the balance between longitudinal growth and structural robustness, which are characteristics directly associated with survival and performance after transplanting [32,33]. The improvement in this parameter suggested that the biostimulants promoted more balanced growth, avoiding both excessive elongation and restricted vegetative development. This response may be explained by the coordinated action of cytokinins, auxins, and gibberellins on cell elongation and stem thickening, in addition to the stimulation of meristematic activity and leaf expansion promoted by bioactive compounds present in the formulations [26,34,35].

With respect to biomass accumulation, the root dry mass values observed in this study were greater than those reported by Hafle et al. [36], which ranged from 1.40 to 4.41 g plant^−1^, and exceeded most of the values obtained by Cruz et al. [37], whose results ranged from 1.17 to 6.72 g plant^−1^, depending on the substrate compositions evaluated. These results demonstrate the high root development capacity of the seedlings produced under the experimental conditions adopted, an aspect of particular importance for species cultivated in semiarid environments, where efficient soil exploration is an essential mechanism for initial plant establishment.

The substantial root development observed was likely associated with favorable growing conditions and the substrate’s adequate nutrient supply, factors that promote the preferential allocation of carbon to the root system during early developmental stages [38,39]. At this stage, root expansion represents a strategic investment to increase water and nutrient uptake capacity, a process frequently prioritized by young plants. Consequently, the lack of a significant response to the biostimulants in terms of root and total biomass suggests that the growth potential of the root system was already near its physiological maximum under the experimental conditions evaluated, thereby limiting the magnitude of any additional effects induced by the plant regulators.

On the other hand, the gradual increase in shoot dry biomass and the shoot-to-root ratio suggests changes in carbon partitioning patterns among plant organs. According to Calvo et al. [40], biostimulants can act by increasing the strength of metabolic sinks, favoring the direction of assimilates toward actively growing tissues. Thus, even without promoting significant increases in total biomass, these products can modify the internal distribution of resources, stimulating vegetative growth and shoot biomass accumulation. This mechanism is consistent with the hypothesis that biostimulants act primarily on the efficiency of photoassimilate utilization and allocation rather than necessarily on the plant’s total carbon production.

The Dickson quality index reinforces this interpretation. The values estimated from the results of Cruz et al. [37] ranged from 0.51–2.43, whereas the data from Hafle et al. [36] yielded estimated values between 0.51 and 1.81. Comparatively, the mean value obtained in the present study (1.96) exceeded most results reported in the literature for nursery-grown *S. tuberosa* seedlings; it was approximately 35% higher than the maximum value estimated by Hafle et al. [36] and close to the highest indices reported by Cruz et al. [37]. Given that these results were obtained without altering the substrate composition, the data indicate that the application of biostimulants contributed to improving the morphophysiological quality of the seedlings, independent of modifications to the root environment.

### 3.2. Photosynthetic and Physiological Responses of Spondias tuberosa Seedlings to the Application of Biostimulants

The leaf chlorophyll levels remained within the range previously reported for *S. tuberosa* by Cavalcante et al. [17], indicating that the application of biostimulants did not cause significant changes in the biosynthesis or stability of photosynthetic pigments. This pattern suggests that, in woody species adapted to semiarid environments, the chlorophyll content tends to be more strongly influenced by nutritional status and cultivation conditions than by the individual application of plant growth regulators or bioactive compounds [17]. The stability of the pigments also demonstrated that the treatments did not induce physiological imbalances capable of compromising the plant’s capacity to capture and utilize light energy.

Similarly, chlorophyll fluorescence variables (Fo, Fm, Fv, Fv/Fo, and Fv/Fm) remained stable across treatments, demonstrating that the biostimulants did not significantly alter the photochemical functioning of photosystem II (PSII). Although the mean Fv/Fm values (0.65) were lower than the classic range reported for nonstressed leaves [41,42], the absence of concomitant changes in growth, biomass production, and gas exchange suggests that this response was not associated with severe photoinhibitory damage. During the evaluations, the high air and leaf temperatures recorded in the greenhouse likely contributed to a moderate reduction in the maximum photochemical efficiency of PSII, given that conditions of high thermal load can induce energy dissipation mechanisms and photoprotective adjustments without necessarily compromising carbon assimilation and plant growth [42,43,44].

Although lower than the classic values reported for nonstressed leaves, the Fv/Fm values were consistent across all the treatments and were not accompanied by reductions in growth, biomass, or net carbon assimilation. This behavior indicates that the observed response likely reflected an environmental condition common to all the evaluated plants, associated with the high temperatures recorded in the greenhouse, rather than a specific effect resulting from the application of the biostimulants.

The gas exchange variables also remained stable across the treatments, indicating that the biostimulants did not significantly alter the diffusive and biochemical components of photosynthesis. These results suggest that growth and quality responses may be associated with adjustments in assimilate partitioning and source–sink dynamics. The most significant finding is that the observed increases in growth, shoot biomass, and seedling quality occurred without changes in stomatal conductance, transpiration, internal CO_2_ concentration, or net carbon assimilation. These results suggest that growth and quality responses may be associated with adjustments in assimilate partitioning and source–sink dynamics rather than with increases in instantaneous photosynthesis [21,45].

According to Rouphael and Colla [21], biostimulants can act through hormonal modulation, improved nutrient-use efficiency, strengthened source–sink relationships, and optimized metabolic processes involved in the utilization of assimilated carbon. Under these conditions, the same photosynthetic rate can support greater biomass production due to increased efficiency in converting assimilates into vegetative structures.

This decoupling of growth from instantaneous photosynthesis has been recognized as one of the most common responses associated with the use of biostimulants. Under these conditions, growth gains do not necessarily stem from higher carbon assimilation rates but rather from greater metabolic efficiency in utilizing the assimilates already produced by the plant. Consequently, a given photosynthetic rate can support increased vegetative growth, greater biomass accumulation, and improved seedling quality, demonstrating that biostimulants may act primarily on postphotosynthetic processes related to carbon partitioning, meristematic activity, and structural development.

Furthermore, bioactive compounds present in biostimulants can stimulate meristematic activity and increase the sink strength of growing tissues, favoring the allocation of resources to leaves, stems, and other metabolically active organs [40,45]. This mechanism may explain the increased growth and seedling quality observed in this study, even in the absence of significant changes in instantaneous photosynthetic variables.

The results highlight the importance of distinguishing between statistically significant effects and descriptive trends. Increases in root biomass, total biomass, and the Dickson quality index were detected via ANOVA (*p* ≤ 0.05) but were not supported by regression models. Conversely, the quadratic patterns observed in the fluorescence variables, which peak at intermediate doses (1.7–1.9 mL L^−1^), should be interpreted solely as potential physiological trends and lack statistical validation. This distinction prevents overinterpretation and lends greater scientific consistency to the conclusions.

Additionally, the stability of the internal CO_2_ concentration, relative stomatal limitation, intrinsic water-use efficiency, and instantaneous carboxylation efficiency indicate that limitations on carbon assimilation are predominantly determined by greenhouse environmental conditions and species ecophysiological characteristics rather than by the applied biostimulant dosages. Recent reviews have demonstrated that the benefits of these inputs are frequently associated with the maintenance of photochemical integrity, metabolic balance, and greater resource-use efficiency, reflected in increased vigor and biomass accumulation without consistent changes in instantaneous gas exchange variables [46,47,48,49].

These results suggest that the growth and quality of *S. tuberosa* seedlings may be associated with mechanisms linked to assimilate partitioning and increased sink strength in growing tissues, without significant changes in instantaneous photosynthetic variables. Increases in root biomass, total biomass, and the Dickson quality index were confirmed by ANOVA but not supported by regression models, whereas quadratic patterns in fluorescence variables should be interpreted merely as potential physiological trends. The stability of the internal CO_2_ concentration and water-use efficiency indicates that limitations on carbon assimilation stemmed primarily from environmental conditions and the species’ ecophysiology. These findings align with recent studies showing growth gains without a direct increase in photosynthesis in tomato seedlings under supplemental lighting [50], papaya plants treated with *Ascophyllum nodosum* extract [51], wheat under salt stress treated with silicon [52], grasses treated with mineral biostimulants [53], and microalgae stimulated by bioactive extracts [54].

From an applied perspective, producing seedlings with enhanced growth and quality without increasing transpiration or reducing photochemical efficiency is important for production systems located in semiarid regions. Under these conditions, maintaining physiological stability alongside increased growth is desirable, as it allows for the production of more vigorous seedlings without a proportional rise in water demand during the nursery phase.

### 3.3. Integrated Growth and Physiological Responses to Biostimulant Application

Principal component analysis (PCA) revealed that leaf temperature played a central role in organizing the physiological and biometric responses of the seedlings, acting as an integrating variable among growth, gas exchange, and photochemical activity. The observed associations between leaf temperature, carbon assimilation, and biometric variables suggest that the primary axis of physiological variation in the seedlings was related to their capacity to maintain growth under conditions of high thermal load. This pattern demonstrates that plant performance results from the coordinated interaction among energy balance, photosynthetic function, and assimilate utilization, reflecting the high capacity of a species for physiological acclimation to the greenhouse environment.

The distribution of variables across the principal components demonstrated that the responses elicited by the biostimulants did not occur in isolation but involved integrated mechanisms linking growth, photosynthetic metabolism, and physiological efficiency. According to Rouphael and Colla [21], biostimulants often act on multiple metabolic pathways simultaneously, promoting systemic responses related to nutrient use efficiency, hormonal modulation, redox balance, and more efficient energy resource utilization. In this context, the principal component analysis reinforces the interpretation derived from the univariate analyses, indicating that the observed growth increases did not depend solely on changes in gas exchange but rather on broader physiological adjustments related to metabolic efficiency and assimilate allocation.

Cluster analysis complemented this interpretation by highlighting the existence of different functional strategies for responding to biostimulants. The resulting groups presented distinct levels of physiological plasticity and metabolic reorganization, reflecting the ability of the seedlings to respond differently to the bioactive compounds present in the formulations. In ecophysiological studies, this pattern is often interpreted as the result of the interaction between the intrinsic characteristics of a species and the stimuli induced by the treatments, leading to varying combinations of growth, physiological maintenance, and resource-use efficiency [48,55].

The first group showed a stronger association with variables related to physiological maintenance and photochemical efficiency, characterizing a more conservative strategy in which available resources are allocated primarily to preserving metabolic function. In contrast, the second group demonstrated a better balance among growth, gas exchange, and chlorophyll fluorescence, suggesting greater physiological plasticity and improved integration between carbon acquisition and vegetative development. This behavior indicates a more efficient use of available physiological resources, enabling growth without a significant increase in the costs associated with transpiration and photosynthetic metabolism [48,56].

In turn, the third grouping, associated primarily with the highest doses and the responses elicited by Bio II, showed greater proximity to variables related to structural growth and biomass accumulation. This pattern suggests heightened physiological responsiveness to the biostimulant, likely resulting from the combined action of plant regulators, amino acids, and other bioactive compounds capable of stimulating meristematic activity and increasing the sink strength of growing tissues. The distinct multivariate separation of this group reinforces the hypothesis of an optimal physiological response range, which is consistent with the concept of hormesis described by Calabrese and Blain [29], wherein metabolic stimulation favors the allocation of assimilates toward growth without compromising photochemical stability or gas exchange.

These results suggest that growth and quality responses may be associated with adjustments in assimilate partitioning and source–sink dynamics rather than with increases in instantaneous photosynthesis. This mechanism helps explain the discrepancy observed between the 4.5 mL L^−1^ dose, which maximized morphological growth, and the intermediate doses (1.7–1.9 mL L^−1^), which favored photochemical performance. Thus, the results suggest that improved photoassimilate utilization and allocation may have contributed to the observed responses, modulating the balance between structural growth and physiological adjustments without significantly altering the instantaneous gas exchange variables. This distinction between growth responses and photochemical responses was also highlighted by multivariate analysis, which enabled the integration of relationships between the treatments and the set of morphophysiological variables evaluated.

Multivariate analysis (PCA and hierarchical clustering) revealed the formation of three distinct clusters, reflecting different patterns of association between the treatments and the evaluated variables. In Cluster 1, the control plants and the plants in the lower-dose treatments were grouped, which was associated with lower growth and biomass values. Cluster 2 was more closely related to chlorophyll fluorescence variables, whereas Cluster 3, comprising mainly treatments with relatively high doses of Bio II, was associated with relatively high values for stem diameter, biomass, and the Dickson quality index. Thus, the arrangement of treatments in multivariate space reinforces the existence of distinct response patterns, in which increases in growth and biomass did not necessarily occur in parallel with changes in photochemical variables. However, these interpretations should be considered exploratory, representing associations observed in multivariate space rather than direct causal relationships between the treatments and the analyzed variables.

**Figure 5:**
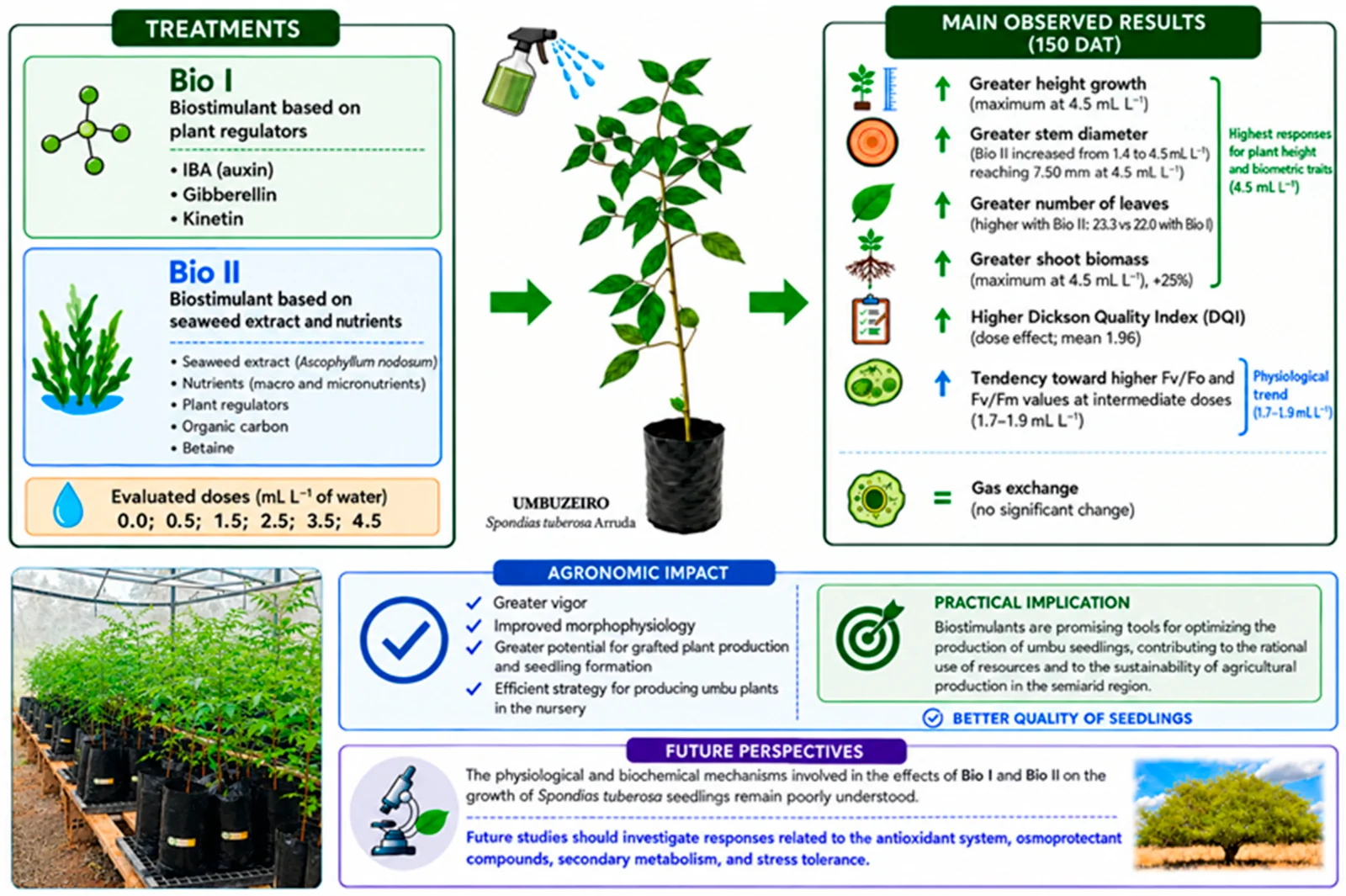
Conceptual model illustrating the effects of biostimulants on the growth and physiological performance of *Spondias tuberosa* Arruda rootstocks. Bio I (S = Stimulate^®^); Bio II (MG = Matriz G^®^).

Integrating these patterns allows for a broader interpretation of plant responses, as the separation of clusters highlights the combined contributions of different components to seedling performance. Taken together, the results of the principal component and cluster analyses suggest that the responses of *S. tuberosa* to biostimulants involve various components of physiological acclimation, wherein growth, thermal regulation, photochemical activity, and assimilate utilization act in an integrated manner. This multivariate approach broadens the understanding of the mechanisms associated with biostimulant action, demonstrating that their effects can extend beyond isolated growth or photosynthetic responses and are related to systemic physiological adjustments that contribute to overall seedling efficiency during nursery establishment. Thus, these results reinforce the potential of biostimulants as a technological tool for optimizing the production of *S. tuberosa* seedlings with superior morphophysiological quality (Figure 5).

Although the results obtained are promising, future studies should evaluate whether the physiological differences observed in nurseries translate into agronomic advantages after transplanting. Assessments involving field survival, early growth, root development, graft compatibility, and responses to water stress conditions could help validate the observed benefits and broaden the understanding of the physiological mechanisms associated with the use of biostimulants in fruit species native to semiarid regions.

## 4. Materials and Methods

**Figure 6:**
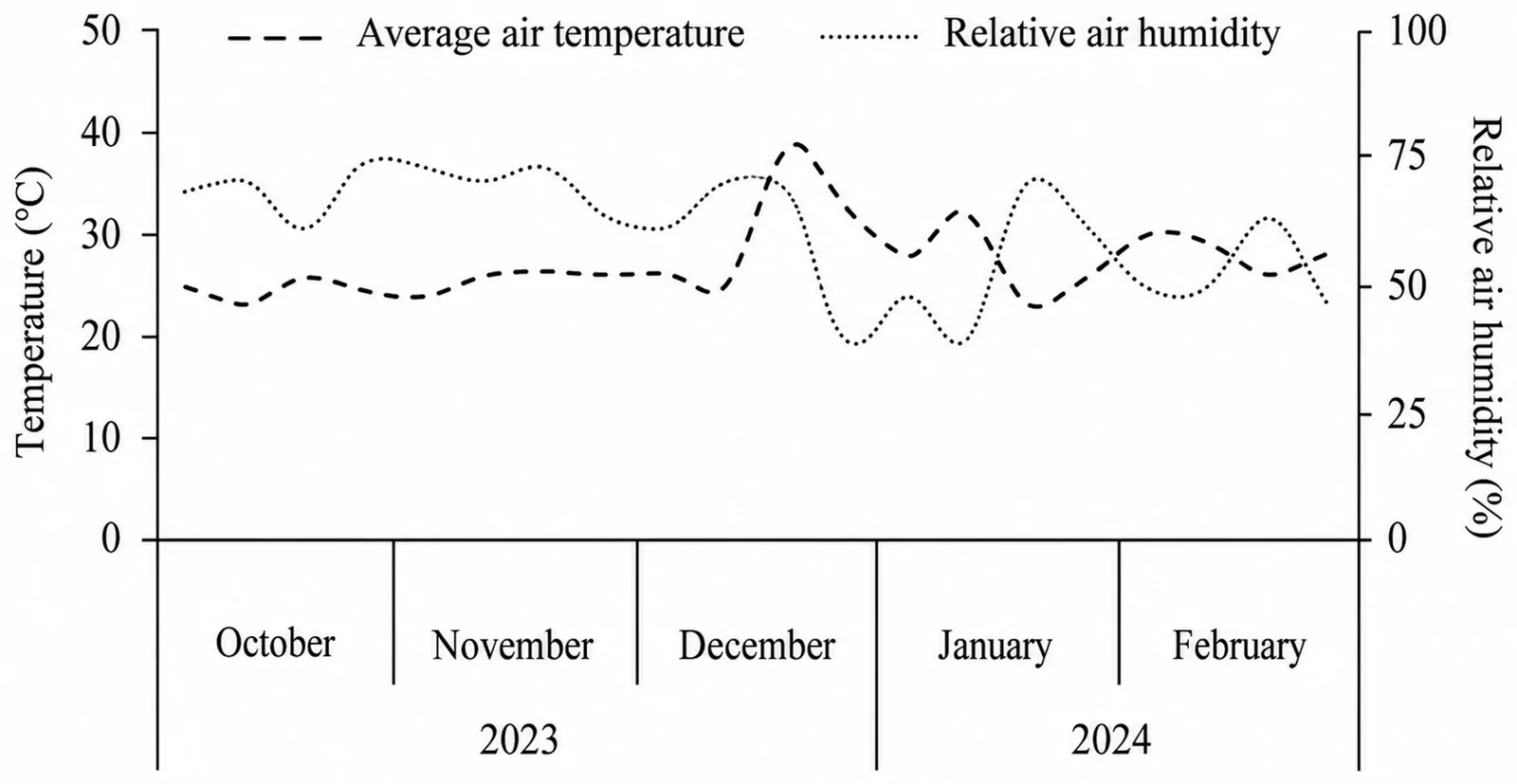
Average air temperature and relative humidity during the experiment with *Spondias tuberosa* Arruda rootstocks.

The experiment was conducted from October 2023 to February 2024 in a screened greenhouse (6°58′4.84″ S, 35°43′0.26″ W; altitude: 557 m) at the Department of Plant Science and Environmental Sciences (DFCA) of the Federal University of Paraíba (UFPB), in the municipality of Areia, Paraíba, Brazil. The atmospheric temperature and relative humidity inside the greenhouse were monitored via an Exbom digital thermohygrometer, model FEPRO-MUT600S, manufactured by the company Exbom, based in the city of São Paulo, Brazil (Figure 6).

### 4.1. Treatments and Experimental Design

The treatments were arranged in a 2 × 6 factorial design, comprising two biostimulant sources (Bio I and Bio II) and six concentrations (0, 0.5, 1.5, 2.5, 3.5, and 4.5 mL L^−1^ of water), totaling 12 treatments (Figure S1). These plants were distributed across three randomized blocks, with each experimental unit consisting of six plants. All the plants within each unit were used for morphological evaluations. Bio-I (Stimulate^®^) contains 50 mg L^−1^ IBA (indole-3-butyric acid—auxin), 50 mg L^−1^ gibberellin, and 90 mg L^−1^ kinetin. Moreover, Bio II (Matriz G^®^) is an organomineral fertilizer containing 13 g L^−1^ N, 26 g L^−1^ K, 13 g L^−1^ Mg, 31.2 g L^−1^ S, 6.5 g L^−1^ B, 6.5 g L^−1^ Mn, 1.3 g L^−1^ Mo, 26 g L^−1^ Zn, 78 g L^−1^ organic carbon, 50 mg L^−1^ auxin, 100 mg L^−1^ cytokinin, 50 mg L^−1^ gibberellin, and 60 mg L^−1^ betaine, with 20% seaweed extract (*Ascophyllum nodosum*).

The concentrations were determined on the basis of the manufacturers’ technical recommendations and ranges reported in preliminary studies of these biostimulants on fruit trees. The 1.0 mL L^−1^ interval allowed for the assessment of gradual responses and the identification of more efficient doses, such as 4.5 mL L^−1^ for growth and intermediate doses for photochemical adjustments.

### 4.2. Research Procedures

**Figure 7:**
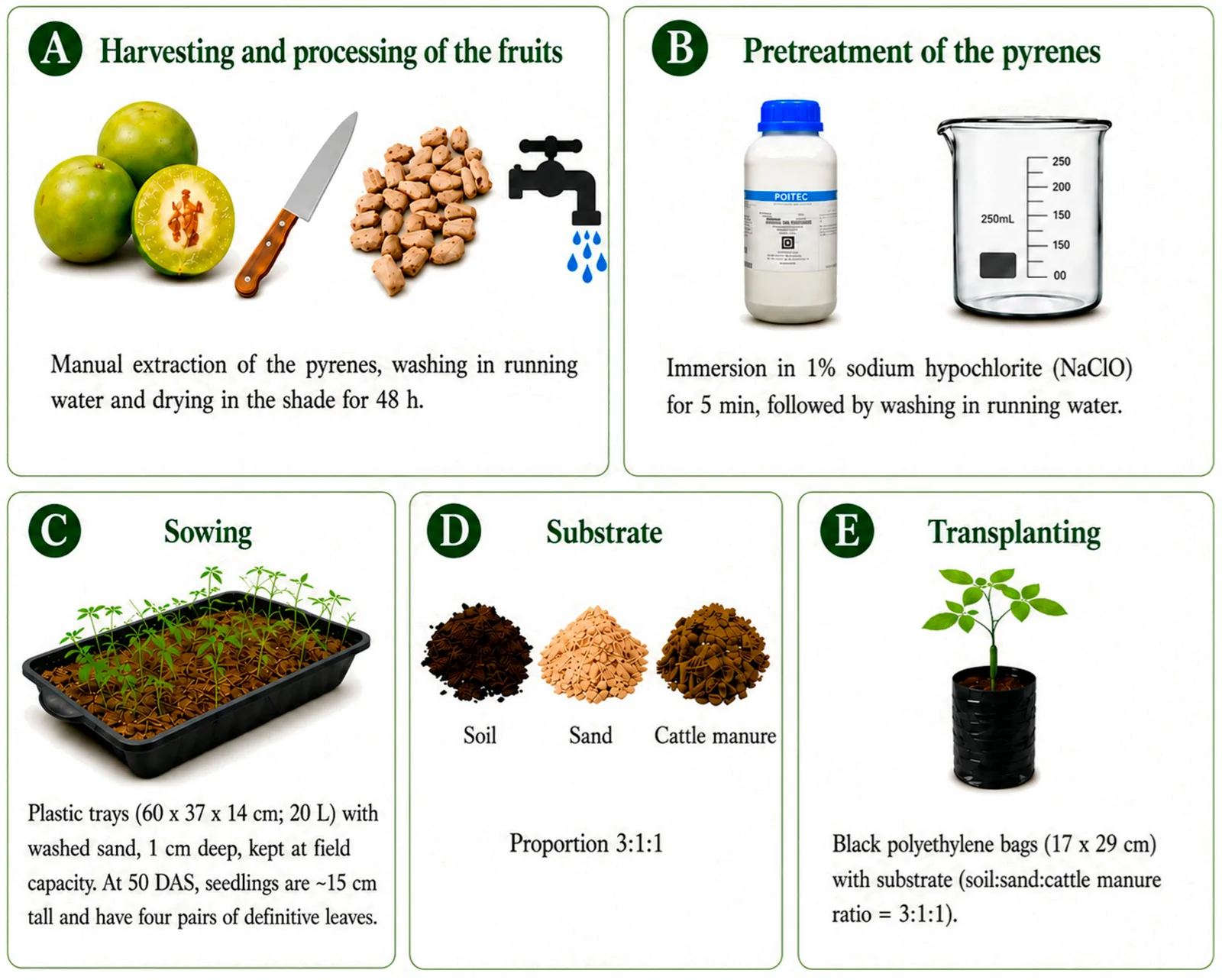
Methodological steps for the production of *Spondias tuberosa* Arruda rootstocks. (**A**) Harvesting and processing of fruits; (**B**) pretreatment of the pyrenes; (**C**) sowing; (**D**) substrate; (**E**) transplanting of seedlings.

The pyrenes used to produce the seedlings were extracted from ripe fruits of *Spondias tuberosa* Arruda collected in June 2023 from a mother plant (6°48′40″ S; 35°54′55″ W) in the municipality of Algodão de Jandaíra, Paraíba state, Brazil. After collection, the fruits were processed manually: the pulp was removed via a stainless steel blade, and the pyrenes were washed in running water to remove residues and then air-dried in the shade for 48 h in a ventilated environment. Subsequently, visual selection was performed, discarding pyrenes with physical damage or signs of predation (Figure 7). Visually healthy and well-formed pyrenes were placed in Ziploc-style plastic bags and stored at 10 °C until sowing.

Prior to sowing, the pyrenes were immersed in a 1% sodium hypochlorite (NaClO) solution for 5 min, followed by washing under running water. Sowing was carried out in plastic trays measuring 60 × 37 × 14 cm (length, width, and depth, respectively) filled with washed sand; the seeds were sown at a depth of 1 cm, and the substrate was maintained at field capacity. At 50 days after sowing (DAS), when the seedlings had reached an average height of 15 cm and developed four pairs of true leaves, they were transplanted into black polyethylene bags measuring 17 × 29 cm (diameter and height, respectively). These containers were filled with a substrate (Table 2) composed of soil, washed sand, and well-developed cattle manure at a 3:1:1 ratio.

Biostimulant applications began 40 days after transplanting via a 500 mL polypropylene sprayer; each plant received approximately 8 mL, covering the entire aerial part. Spraying was performed weekly throughout the experimental period at 7:00 AM, totaling 20 applications. The seedlings were irrigated twice a week to maintain substrate moisture at approximately 60% of field capacity [57]. The field capacity (FC) was determined from four substrate samples via the following formula:

$$\mathrm{FC}\left(g{\;g}^{-1}\right)=\frac{\left(\mathrm{Mass of wet sample}-\mathrm{Mass of dry sample}\right)}{\mathrm{Mass of wet sample}}\times 100$$

Following this step, the samples were taken to the greenhouse, where the experiment was conducted, and weighed daily to determine the water retention curve of the substrate.

### 4.3. Evaluations

At 150 days after transplanting (DAT), the following parameters were evaluated: plant height (PH, in cm), measured with a tape measure from the substrate level to the tip of the orthotropic branch [58]; stem diameter at the substrate level (SD, in mm), measured with a digital caliper (MTX-316119) from the brand MTX, manufactured in Shanghai, China; number of leaves (NL), determined by counting fully expanded leaves; and shoot dry mass (SDM), root dry mass (RDM), and total dry mass (TDM). For these measurements, the plant parts were separated, placed in Kraft paper bags, oven-dried at 65 °C for 72 h, and weighed, and the results are expressed in grams. The dry biomass distribution between the shoot and roots (SDM/RDM) and the Dickson quality index (DQI) [59] were calculated via the following equation:

$$\mathrm{DQI}=\frac{\mathrm{TDM}}{\frac{\mathrm{PH}}{\mathrm{SD}}+\frac{\mathrm{SDM}}{\mathrm{RDM}}}$$

where:

DQI—Dickson Quality Index;
TDM—Total dry matter mass (g);
PH—Plant height (cm);
SD—Stem diameter at the substrate level (mm);
SDM—Shoot dry matter mass (g);
RDM—Root dry matter mass (g).

Physiological assessments were conducted in the morning on the same leaflet, specifically at the mid-section of the leaf [58]. Leaf chlorophyll indices for ‘a’ (ICLa), ‘b’ (ICLb), and total (ICLtotal), as well as the ‘a’/‘b’ ratio (ICLa/ICLb), were measured via a portable Falker^®^ CFL 1030 chlorophyll meter on four plants per experimental plot. The initial (Fo), maximum (Fm), and variable (Fv) fluorescence; the ratio of photochemical to nonphotochemical processes (Fv/Fo); and the quantum efficiency of photosystem II (Fv/Fm) were measured via a portable Opti-Sciences^®^ OS-30P fluorometer (manufactured by Opti-Sciences in Hudson, New Hampshire, USA) on two plants per experimental plot, following a 30-min dark adaptation period using leaf clips.

Gas exchange parameters, including leaf temperature (T, °C), stomatal conductance (Gs, mmol H_2_O m^−2^ s^−1^) and resistance (Rs, s mol^−1^ H_2_O m^−2^), internal CO_2_ concentration (Ci, μmol CO_2_ mol^−1^ air), relative stomatal limitation (Ci/Ca), net CO_2_ assimilation (A, μmol CO_2_ m^−2^ s^−1^), transpiration (E, mmol H_2_O m^−2^ s^−1^), water-use efficiency (WUE, μmol CO_2_ mol^−1^ H_2_O), intrinsic water-use efficiency (iWUE, μmol CO_2_ mol^−1^ H_2_O), and carboxylation efficiency (CE, mol CO_2_ m^−2^ s^−1^), were determined via an ADC BioScientific^®^ LCpro-SD portable photosynthesis analyzer-IRGA (manufactured by ADC BioScientific Ltd., based in Hoddesdon, United Kingdom) on two plants per experimental plot under a radiation level of 1500 µmol photons m^−2^ s^−1^ and an ambient CO_2_ concentration of 400 µmol CO_2_ mol^−1^ air. 

### 4.4. Statistical Analysis of the Data

Initially, the assumptions for the analysis of variance were evaluated, specifically normality (Anderson–Darling, *p* ≤ 0.05) and homogeneity of variances (Bartlett and Levene, *p* ≤ 0.05), on the basis of the model residuals. The data were subsequently subjected to analysis of variance (ANOVA, *p* ≤ 0.05) to determine the significance of the main effects and factor interactions. Contrasts (F test, *p* ≤ 0.05) were performed on the means of biofertilizer sources, whereas polynomial regression fits (*p* ≤ 0.10) were tested for concentrations; regression analysis was applied to each source when the source effect or interaction was significant or to the means across sources when neither the source effect nor the interaction was significant. Multivariate analyses were conducted via principal component analysis (PCA) and cluster analysis (CA) on the basis of treatment scores [60,61]. All analyses were performed via R^®^ software version 4.4.0 (R Core Team 2024).

ŷ*_ijk_* = *μ* + *F_i_* + *C_j_* + (*F* × *C*)*_ij_* + *e_ijk_*

## 5. Conclusions

Foliar application of biostimulants (Bio I and Bio II) promotes increases in vegetative growth and seedling quality in *Spondias tuberosa*, highlighting their potential for producing seedlings intended for orchard establishment and rootstock production.

Increasing the biostimulant dosage promoted height growth, shoot biomass accumulation, and an improvement in the Dickson quality index, with the highest responses recorded for plant height and specific biometric traits at 4.5 mL L^−1^ within the evaluated concentration range.

Gas exchange was not significantly altered, indicating physiological stability. Intermediate doses (1.7–1.9 mL L^−1^) tended to increase the Fv/Fo and Fv/Fm values; however, no significant treatment effect was detected. These responses may suggest adjustments in the photosynthetic apparatus, although this mechanism was not directly evaluated in the present study.

Multivariate analysis revealed associations among growth characteristics, photochemical activity, and leaf temperature in *S. tuberosa*, highlighting leaf temperature as an important variable in the integration of the responses of the physiological apparatus evaluated.

The results demonstrate that the application of biostimulants is a promising strategy to accelerate the production of *Spondias tuberosa* seedlings, contributing to the development of higher-quality rootstocks, the establishment of commercial orchards, and the advancement of programs for the domestication and valorization of this important species native to the Brazilian Caatinga.

Although the physiological indicators evaluated did not significantly differ, the increases in height and biomass indicate that the biostimulants acted primarily on the partitioning of assimilated carbon, prioritizing growth while maintaining the physiological stability of the seedlings. This stability suggests that the products promote growth without drastically altering basic physiological processes, which is desirable for vigorous rootstocks.

Future studies should investigate the persistence of these effects after transplanting, as well as their impacts on the establishment, early field growth, and performance of seedlings subjected to water stress conditions, thereby broadening the understanding of the physiological mechanisms associated with the use of biostimulants in fruit species adapted to semiarid regions.

## Figures and Tables

**Table 1:** Matrix of eigenvalues and eigenvectors of the principal components (PCs) of morphophysiological variables in *Spondias tuberosa* Arruda rootstocks.

	PC1	PC2	PC3	PC4
Eigenvalue (λ)	7.90	6.63	4.02	3.17
Explained variance (%)	28.21	23.68	14.36	11.32
Cumulative variance (%)	28.21	51.89	66.25	77.57
	Eigenvector ^1^ (%)			
PH	**0.2507**	−0.1186	−0.1426	0.2178
SD	−0.0673	0.1072	−0.0624	**0.2934**
NL	**0.2593**	−0.0780	−0.0570	−0.1293
PH/SD	**0.2495**	−0.1674	−0.0880	0.0516
RDM	0.1623	**0.2848**	0.0012	0.2627
SDM	0.1352	0.1813	−0.0689	**0.4290**
SDM/RDM	−0.0702	−0.1780	−0.1692	**0.3399**
TDM	0.1609	**0.2710**	−0.0111	**0.2977**
DQI	0.0241	**0.3036**	0.0701	0.2702
Chl a	−0.0546	**0.2512**	−0.1763	−0.1973
Chl b	0.1104	**0.2513**	**−0.2565**	−0.1836
Chl total	0.0437	**0.2709**	−0.2396	−0.2045
Chl a/Chl b	−0.1265	**−0.2184**	**0.2613**	0.2002
Fo	−0.1401	−0.1000	−0.1018	0.1491
Fm	0.1513	0.0123	**0.3600**	0.0707
Fv	0.1706	0.0460	**0.3870**	0.0074
Fv/Fo	**0.2160**	0.0591	**0.3692**	−0.0994
Fv/Fm	0.1610	0.0255	**0.3937**	−0.1771
Tleaf	**−0.3129**	−0.0172	−0.0724	−0.0863
Gs	**0.2312**	−0.1475	−0.1676	−0.0794
Rs	−0.1300	**0.3173**	0.0508	−0.0983
Ci	**0.3092**	0.0893	−0.0863	0.0194
Ci/Ca	**0.3101**	0.0828	−0.0901	0.0278
A	−0.0288	**−0.2612**	−0.1790	0.1402
E	**0.2407**	−0.1776	−0.0650	−0.0582
WUE	**−0.2249**	**0.2327**	0.0769	0.0613
CE	**−0.1918**	−0.1300	0.1535	0.2022
iWUE	**−0.2091**	**0.2388**	0.0743	0.0388

^1^ The criterion used to separate the variables representing the component was based on values exceeding the absolute value of the expression 0.5/(λ^0.5^), with such values highlighted in bold [24]. **Abbreviations**: PH, plant height; SD, stem diameter at substrate level; NL, number of leaves; PH/SD, plant height-to-stem diameter ratio; RDM, root dry matter mass; SDM, shoot dry matter mass; SDM/RDM, shoot-to-root dry matter mass ratio; TDM, total dry matter mass; DQI, Dickson Quality Index; Chl a, chlorophyll a; Chl b, chlorophyll b; Chl total, total chlorophyll; Chl a/Chl b, chlorophyll a-to-b ratio; F_0_, initial fluorescence; Fm, maximum fluorescence; Fv, variable fluorescence; Fv/F_0_, ratio between photochemical and nonphotochemical processes; Fv/Fm, quantum efficiency of photosystem II; Tleaf, leaf temperature; gs, stomatal conductance; rs, stomatal resistance; Ci, intercellular CO_2_ concentration; Ci/Ca, relative stomatal limitation; A, net CO_2_ assimilation; E, leaf transpiration; WUE, water-use efficiency; CE, carboxylation efficiency; iWUE, intrinsic water-use efficiency.

**Table 2:** Chemical (fertility) and physical attributes of the substrate used for the production of *Spondias tuberosa* seedlings.

Chemical Attributes (Fertility)											
pH	P	K^+^	Na^+^	Ca^2+^	Mg^2+^	SB	H^+^ + Al^3+^	CTC	V	ESP	OM
^______^	mg dm^−3^	^______________________^ cmol_c_ dm^−3 ______________________^							^______^ % ^______^		g kg^−1^
7.2	260.33	1.00	0.24	4.79	1.94	7.97	0.41	8.38	95.11	2.86	24.74
Physical Attributes											
Sand		Silt				Clay			Textural Class		
^__________________________________^ g kg^−1 __________________________________^											
862		104				34			Areia Franca		

pH (hydrogen potential) in water; P (phosphorus), K^+^ (potassium), and Na^+^ (sodium) extracted with Mehlich-1; Ca^2+^ (calcium), Mg^2+^ (magnesium), and Al^3+^ (aluminum) extracted with 1 M KCl; H^+^ + Al^3+^ (hydrogen plus aluminum) extracted with 0.5 M calcium acetate at pH 7.0; SB (sum of bases) = K^+^ + Na^+^ + Ca^2+^ + Mg^2+^; CEC (cation exchange capacity) = SB + H^+^ + Al^3+^; V (base saturation) = (SB/CEC) × 100; ESP (exchangeable sodium percentage) = (Na^+^/CEC) × 100; OM (organic matter) = organic carbon × 1.724, using the Walkley–Black method. Particle-size distribution was determined using the hydrometer method, with 1 M NaOH as the dispersing agent.

## Data Availability

The original contributions presented in this study are included in the article. Further inquiries can be directed to the corresponding author.

## Supplementary Materials

The following supporting information can be downloaded at: https://www.mdpi.com/article/10.3390/plants15213351/s1, Figure S1: Photographic record of the *Spondias tuberosa* Arruda seedlings subjected to all treatments with the two biostimulants (Bio I = Stimulate^®^ and Bio II = Matriz G.

## References

[1] Mertens J., Germer J., Siqueira Filho J.A., Sauerborn J. (2017). *Spondias tuberosa* Arruda (Anacardiaceae), a threatened tree of the Brazilian Caatinga?. Braz. J. Biol..

[2] Ferreira S.V.F., Campos A.R.N., Medeiros M.F.T. (2019). Análise prospectiva da espécie-chave cultural umbuzeiro (*Spondias tuberosa* Arr.) do Semiárido Brasileiro. Cad. Prospecção.

[3] Lorenzi H. (1998). Árvores Brasileiras: Manual de Identificação e Cultivo de Plantas Arbóreas Nativas do Brasil.

[4] Nascimento-Silva O., Paiva J.G.A. (2007). Estudos morfológicos e anatômicos em folhas adultas de *Spondias tuberosa* Arruda (Anacardiaceae). Bol. Latinoam. Caribe Plan. Med. Aromáticas.

[5] Lima Filho J.M.P. (2011). Ecofisiologia do Umbuzeiro (Spondias tuberosa Arruda Câmara).

[6] Lins-Neto E.M.F., Peroni N., Maranhão C.M.C., Maciel M.I.S., Albuquerque U.P. (2012). Analysis of umbu (*Spondias tuberosa* Arruda (Anacardiaceae)) in different landscape management regimes: A process of incipient domestication. Environ. Monit. Assess..

[7] Costa F.R., Santos C.A.F., Lima R.M.S., Oliveira V.R. (2015). Análise biométrica de frutos de umbuzeiro do semiárido brasileiro. Biosci. J..

[8] Silva A.R.A., Morais S.M., Marques M.M.M., Oliveira D.F., Barros C.C., Almeida R.R., Vieira Í.G.P., Guedes M.I.F. (2012). Chemical composition, antioxidant and antibacterial activities of two Spondias species from Northeastern Brazil. Pharm. Biol..

[9] Zeraik M.L., Queiroz E.F., Marcourt L., Ciclet O., Castro-Gamboa I., Silva D.H.S., Cuendet M., Bolzani V.S. (2016). Antioxidants, quinone reductase inducers and acetylcholinesterase inhibitors from *Spondias tuberosa* fruits. J. Funct. Foods.

[10] Mitchell J.D., Daly D.C. (2015). A revision of *Spondias* L. (Anacardiaceae) in the Neotropics. PhytoKeys.

[11] Brasil Ministério do Meio Ambiente (2017). Umbu: Boas Práticas Para o Extrativismo Sustentável Orgânico.

[12] Companhia Nacional De Abastecimento—Conab (2026). Boletim Trimestral Sociobiomais Umbu (*Spondias tuberosa*). https://www.gov.br/conab/pt-br/atuacao/sociobiodiversidade/boletins/boletins-2o-trimestre/boletim_umbu_2_trimestre_2026.pdf/view.

[13] Donato S.L.R., Gonçalves N.P., Santos L.J.S., Saturnino H.M., Arantes A.M., Londe L.C., Cardoso M.M. (2019). Prospecção e avaliação de acessos de umbuzeiro. Inf. Agropecuário.

[14] Pires E.S., Amaro C.L., Freitas I.A.S., Lima G.H.F., Ganem E.O., Matos F.S. (2020). Análise de crescimento de plantas de umbuzeiro sob diferentes concentrações de giberelina. Agrarian.

[15] Batista F.R.C., Silva S.M., Santana M.F.S., Cavalcante A.R. (2015). O Umbuzeiro e o Semiárido Brasileiro.

[16] De Araujo F.P., Santos C.A.F., Cavalcanti N.D.B. (2000). Cultivo do Umbuzeiro.

[17] Cavalcante A.C.P., Cavalcante A.G., Henrique J.D.S., Zuza J.F.C., Dantas M.M.M., Araujo R.D.C. (2018). Organic substrate and organic foliar fertilizer effect on chlorophyll index and growth in Umbuzeiro seedlings. Agron. Colomb..

[18] Grossnickle S.C., Macdonald J.E. (2018). Seedling Quality: History, Application, and Plant Attributes. Forests.

[19] Guimarães Z.T.M., Silva D.C., Ferreira M.J. (2024). Seedling quality and short-term field performance of three Amazonian forest species as affected by site conditions. iForest.

[20] Yakhin O.I., Lubyanov A.A., Yakhin I.A., Brown P.H. (2017). Biostimulants in plant science: A global perspective. Front. Plant Sci..

[21] Rouphael Y., Colla G. (2020). Biostimulants in agriculture. Front. Plant Sci..

[22] Reis J.M.R., Rodrigues J.F., Reis M.A. (2016). Doses e formas de aplicação de bioestimulante na produção de mudas de maracujazeiro. Cult. Agronômica.

[23] Machado R.A., Cayres W.C., Vieira C.V., Val B., Oliveira P., Souza W.J.O., Sanches A.G. (2023). Doses de bioestimulante vegetal no desenvolvimento inicial da soja. Tecnologia e Inovação na Agricultura: Aplicação, Produtividade e Sustentabilidade em Pesquisa.

[24] Collins M.E., Ovalles F.A. (1988). Variability of Northwest Florida Soils by Principal Component Analysis. Soil Sci. Soc. Am. J..

[25] Mäkilä R., Wybouw B., Smetana O., Vainio L., Solé-Gil A., Lyu M., Ye L., Wang X., Siligato R., Jenness M.K. (2023). Gibberellins promote polar auxin transport to regulate stem cell fate decisions in cambium. Nat. Plants.

[26] Ding W., Wang C., Mei M., Li X., Zhang Y., Lin H., Li Y., Ma Z., Han J., Song X. (2024). Phytohormones involved in vascular cambium activity in woods: Current progress and future challenges. Front. Plant Sci..

[27] Martínez-Lorente S.E., Martí-Guillén J.M., Pedreño M.Á., Almagro L., Sabater-Jara A.B. (2024). Higher Plant-Derived Biostimulants: Mechanisms of action and their role in mitigating plant abiotic stress. Antioxidants.

[28] Du Jardin P. (2015). Plant biostimulants: Definition, concept, main categories and regulation. Sci. Hortic..

[29] Calabrese E.J., Blain R.B. (2009). Hormesis and plant biology. Environ. Pollut..

[30] Matos F.S., Freitas I.A.S., Pereira V.L.G., Pires W.K.L. (2020). Effect of gibberellin on growth and development of *Spondias tuberosa* seedlings. Rev. Caatinga.

[31] Shirvani-Naghani S., Fallah S., Pokhrel L.R. (2025). Hormesis in field-grown *Glycine Max* (L.) with Foliar-Treatment of Copper Oxide Nanoparticles Observed in Aerial and Belowground Traits Under Drought Stress. J. Plant Growth Regul..

[32] Avelino N.R., Schilling A.C., Dalmolin Â.C., Santos M.S., Mielke M.S. (2021). Alocação de biomassa e indicadores de crescimento para a avaliação da qualidade de mudas de espécies florestais nativas. Ciênc. Florest..

[33] Schwartz G., Rocha T.S., Almeida M.J.S., Dionisio L.F.S., Corvera R. (2022). Seedlings quality of *Bertholletia excelsa* bonpl. (Lecythidaceae) produced in forest nursery. J. Agric. For..

[34] Ritonga F.N., Zhou D., Zhang Y., Song R., Li C., Li J., Gao J. (2023). The Roles of Gibberellins in Regulating Leaf Development. Plants.

[35] Cunha Neto A.R., Ambrósio A.S., Resende A.J.R., Santos B.R., Nadal M.C. (2025). From Cell Division to Stress Tolerance: The versatile roles of cytokinins in plants. Phyton.

[36] Hafle O.M., Lima Filho P., Rodrigues M.H.B.S., Queiroga R.A., Ferreira Neto J., Santos V.M. (2019). Produção de mudas de umbuzeiro submetidas a diferentes substratos orgânicos. Rev. Agroecol. Semiárido.

[37] Cruz F.R.S., Andrade L.A., Feitosa R.C. (2016). Produção de mudas de umbuzeiro em diferentes substratos e tamanhos de recipientes. Ciênc. Florest..

[38] Marschner P. (2012). Marschner’s Mineral Nutrition of Higher Plants.

[39] Taiz L., Møller I.M., Murphy A., Zeiger E. (2017). Fisiologia e Desenvolvimento Vegetal.

[40] Calvo P., Nelson L., Kloepper J.W. (2014). Agricultural uses of plant biostimulants. Plant Soil Dordr..

[41] Demmig B., Björkman O. (1987). Photon yield of O2 evolution and chlorophyll fluorescence characteristics at 77 K among vascular plants of diverse origins. Planta.

[42] Murchie E.H., Lawson T. (2013). Chlorophyll fluorescence analysis: A guide to good practice and understanding some new applications. J. Exp. Bot..

[43] Butler W.L. (1978). Energy distribution in the photochemical apparatus of photosynthesis. Annu. Rev. Plant Physiol..

[44] Jiao Q., Hu X. (2025). Recent Advances and Emerging Trends in Chlorophyll Fluorescence Parameter Fv/Fm. Phyton.

[45] Colla G., Rouphael Y., Canaguier R., Sveistrup T., Perrone D. (2015). Protein hydrolysates as biostimulants in horticulture. Sci. Hortic..

[46] Baltazar M., Correia S., Guinan K.J., Sujeeth N., Bragança R., Gonçalves B. (2021). Recent Advances in the Molecular Effects of Biostimulants in Plants: An Overview. Biomolecules.

[47] Ma Y., Freitas H., Dias M.C. (2022). Strategies and prospects for biostimulants to alleviate abiotic stress in plants. Front. Plant Sci..

[48] Asif A., Ali M., Qadir M., Karthikeyan R., Singh Z., Khangura R., Di Gioia F., Ahmed Z.F.R. (2023). Enhancing crop resilience by harnessing the synergistic effects of biostimulants against abiotic stress. Front. Plant Sci..

[49] Afonso S., Oliveira I., Guedes F., Meyer A.S., Gonçalves B. (2024). Glycine betaine and seaweed-based biostimulants improved leaf water status and enhanced photosynthetic activity in sweet cherry trees. Front. Plant Sci..

[50] Song J., Zhang R., Yang F., Wang J., Cai W., Zhang Y. (2024). Nocturnal LED Supplemental Lighting Improves Quality of Tomato Seedlings by Increasing Biomass Accumulation in a Controlled Environment. Agronomy.

[51] Ferreira T.R., Rodrigues J.S., Galote J.K.B., Crasque J., Neto B.C., Falqueto A.R., Arantes L.O., Arantes S.D. (2025). Mitigation of High Temperatures with Ascophyllum nodosum Biostimulants in Papaya (*Carica papaya* L.) Seedlings. Plants.

[52] Tobiasz-Salach R., Stadnik B., Mazurek M., Buczek J., Leszczyńska D. (2024). Foliar Application of Silicon Influences the Physiological and Epigenetic Responses of Wheat Grown Under Salt Stress. Int. J. Mol. Sci..

[53] Sosnowski J., Truba M., Malinowska E., Kifer P., Krasnodębski P. (2025). Effect of Tytanit and Stymjod on *Phleum pratense* L. Photosynthetic Activity and the Content of Chlorophyll Pigments. Plants.

[54] Popa D.G., Triteano N., Georgescu F., Lupu C., Shaposhnikov S., Constantinescu-Aruxandei D., Oancea F. (2025). SL-6 Mimic Is a Biostimulant for Chlorella sorokiniana and Enhances the Plant Biostimulant Effect of Microalgal Extract. Plants.

[55] Baghdadi A., Della Lucia M.C., Borella M., Bertoldo G., Ravi S., Zegada-Lizarazu W., Chiodi C., Pagani E., Hermans C., Stevanato P. (2022). A dual-omics approach for profiling plant responses to biostimulant applications under controlled and field conditions. Front. Plant Sci..

[56] Kaushal P., Ali N., Saini S., Pati P.K., Pati A.M. (2023). Physiological and molecular insight of microbial biostimulants for sustainable agriculture. Front. Plant Sci..

[57] Silva E.B., Gonçalves N.P., Pinho P.J. (2005). Limitações nutricionais para crescimento de mudas de umbuzeiro em Latossolo Vermelho distrófico no Norte de Minas. Acta Sci. Agron..

[58] Aidar S.T., Araujo F.P., Chaves A.R.M. (2013). Crescimento Inicial e Trocas Gasosas de Mudas de Spondias Enxertadas Sobre Umbuzeiro.

[59] Dickson A., Leaf A.L., Hosner J.F. (1960). Quality appraisal of white spruce and white pine seedling stock in nurseries. For. Chron..

[60] Silva A.R. (2016). Métodos de Análise Multivariada em R.

[61] Artes R., Barroso L.P. (2023). Multivariate Methods of Statistical Analysis.

