# Supplementary material for: Biostimulants in the Formation of Spondias tuberosa Rootstocks
Source: Plants (Basel). 2026 Oct 29;15(21):3351. doi: 10.3390/plants15213351 (PMC13766165; doi:10.3390/plants15213351)
Supplement: Supplementary file 1 [file plants-15-03351-s001.zip › plants-4545993-supplementary.pdf]

Supplementary Figure

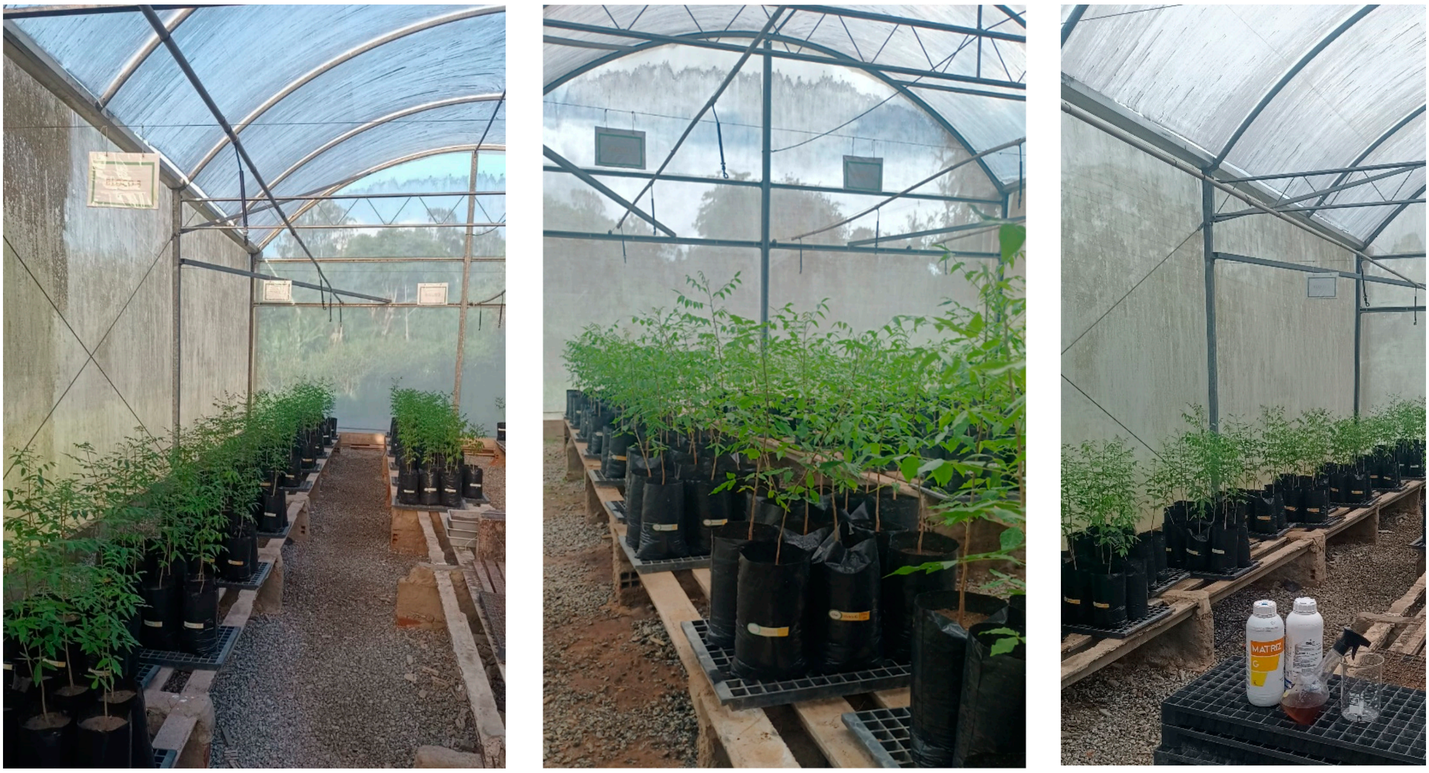

Figure S1. Photographic record of the *Spondias tuberosa* Arruda seedlings subjected to all treatments with the two biostimulants (Bio I = Stimulate® and Bio II = Matriz G®).
